# Nanomechanical and Nanotribological Properties of Nanostructured Coatings of Tantalum and Its Compounds on Steel Substrates

**DOI:** 10.3390/nano11092407

**Published:** 2021-09-15

**Authors:** Galina Melnikova, Tatyana Kuznetsova, Vasilina Lapitskaya, Agata Petrovskaya, Sergei Chizhik, Anna Zykova, Vladimir Safonov, Sergei Aizikovich, Evgeniy Sadyrin, Weifu Sun, Stanislav Yakovin

**Affiliations:** 1 Laboratory of Nanoprocesses and Technologies, A.V. Luikov Heat and Mass Transfer Institute of the National Academy of Sciences of Belarus, 15 P. Brovki Str., 220072 Minsk, Belarus; galachkax@gmail.com (G.M.); kuzn06@mail.ru (T.K.); vasilinka.92@mail.ru (V.L.); agata.petrovskaya@gmail.com (A.P.); chizhik_sa@tut.by (S.C.); 2 National Science Center “Kharkov Institute of Physics and Technology”, 1 Akademicheskaya Str., 61108 Kharkov, Ukraine; zykova.anya@gmail.com (A.Z.); safonov600@gmail.com (V.S.); 3 V.N. Karazin Kharkiv National University, 4 Svobody Sq., 61022 Kharkov, Ukraine; stanislav.yakovin@gmail.com; 4 Research and Education Center “Materials”, Don State Technical University, 1 Gagarin Sq., 344003 Rostov-on-Don, Russia; 5 State Key Laboratory of Explosion Science and Technology, Beijing Institute of Technology, Beijing 100081, China; weifu.sun@bit.edu.cn; 6 Beijing Institute of Technology Chongqing Innovation Center, Chongqing 401120, China

**Keywords:** atomic force microscopy, friction coefficient, magnetron sputtering, nanoindentation, nanostructured coatings, tantalum

## Abstract

The present paper addresses the problem of identification of microstructural, nanomechanical, and tribological properties of thin films of tantalum (Ta) and its compounds deposited on stainless steel substrates by direct current magnetron sputtering. The compositions of the obtained nanostructured films were determined by energy dispersive spectroscopy. Surface morphology was investigated using atomic force microscopy (AFM). The coatings were found to be homogeneous and have low roughness values (<10 nm). The values of microhardness and elastic modulus were obtained by means of nanoindentation. Elastic modulus values for all the coatings remained unchanged with different atomic percentage of tantalum in the films. The values of microhardness of the tantalum films were increased after incorporation of the oxygen and nitrogen atoms into the crystal lattice of the coatings. The coefficient of friction, CoF, was determined by the AFM method in the “sliding” and “plowing” modes. Deposition of the coatings on the substrates led to a decrease of CoF for the coating-substrate system compared to the substrates; thus, the final product utilizing such a coating will presumably have a longer service life. The tantalum nitride films were characterized by the smallest values of CoF and specific volumetric wear.

## 1. Introduction

The stainless steel (for example, 316L SS), platinum iridium alloys, tantalum, nitinol, cobalt–chrome alloys, titanium and its alloys, and pure iron and magnesium alloys are the basic materials for the production of stents. Stainless steel is the most common material for the production of stents with and without coatings. The stents made of stainless steel demonstrate suitable mechanical properties and excellent corrosion resistance. However, the clinical application of steel is limited by the ferromagnetic nature of the alloy and its low density. Due to these properties, the steel is poorly visible in X-ray and magnetic resonance imaging [1]. The implants of the stainless steel may cause an allergy to nickel, chromium, and molybdenum, leading to local immune responses and inflammation. Different materials are used as coatings on stainless steel stents that lead to improvements of X-ray visibility and biocompatibility. Titanium and its alloys have excellent biocompatibility, high corrosion resistance, and are intensively used in orthopedics and dentistry. Pure titanium is not suitable for the stent production due to rather low values of the mechanical properties; however, it can be used as a coating for stainless steel stents to improve biocompatibility. Such stents show excellent results in clinical trials [1,2].

Usage of stents that represent a system of the stainless steel substrate with titanium or tantalum coating allows combining the suitable mechanical properties and bioinertness [3,4,5]. Tantalum is characterized by a good plasticity, high strength, wear resistance, weldability, corrosion resistance, infusibility, biocompatibility, and it is clearly visible in X-rays and magnetic resonance imaging [6,7]. In addition, due to its properties, tantalum is widely used not only in electronics [8,9], protective coatings [10,11], anti-corrosion coatings [12,13], optical coatings [14,15,16,17], chemical industry [18], but also in biomedicine [19,20,21,22,23,24] (orthopedics and dentistry [25,26,27,28,29], endovascular stents, and neurosurgical implants [30]). However, the use of tantalum is difficult due to its high density, manufacturing complexity, and the relatively high cost. For these reasons, a various approaches are currently being proposed to modify the surface properties of metal substrates for the improvement of biological responses by applying coatings based on Ta. The modern processing methods allow obtaining the tantalum coatings with a fine-grained structure and optimal properties (tensile strength up to 600 MPa, and elongation of about 30%) for the stents production [31]. In combination with increased strength, tantalum has a high protective ability that prevents active corrosion processes and the electrochemical destruction of metal surface structures in various environments. For example, β-Ta nanocrystalline coatings on Ti-6Al-4V substrates showed high hardness in combination with good resistance to contact damage [32]. Thus, the corrosion resistance of 316 L stainless steel was significantly improved due to the TaC_x_N_1–x_ coatings [33]. These coatings also demonstrated good adhesion characteristics. Thus, depending on the formation conditions, the properties of the coatings change. However, the best conditions for tantalum coatings formation have not yet been determined.

The development of technologies for the formation of functional tantalum nanostructured coatings is currently of high interest for both medical and material science community [34]. The perspective ion–plasma spraying method for creating nanostructured coatings allows to form nanocoatings to change surface properties and obtain biocompatible materials with desired properties. Thus, in [35], it was found that the TaNx coating applied by high-frequency magnetron sputtering at a relatively high bias voltage of 200 V demonstrates good tribological characteristics, hardness, and adhesive strength. The authors of [36] synthesized tantalum nitride films onto silicon using magnetron sputtering (the N_2_ content in the gas mixture was changed). As a result, it was revealed which content of N_2_ allows obtaining films with the highest hardness, low friction coefficient, and low wear rate. In the present research, a new efficient technology has been developed for the deposition of nanostructured coatings based on tantalum by magnetron sputtering. The optimal modes of metal substrates modification with nanostructured coatings based on Ta were developed in order to create new improved devices for medical applications (cardiovascular surgery, endoscopy, and orthopedics). Remarkably, nanostructured materials have larger surface energy than typical materials enhancing the adhesion of bone cells and producing higher osseointegration [32]. The surface nature of a biomaterial (relief, hydrophobic–hydrophilic properties, chemical composition, etc.) plays an important role in regulating the cellular response of a biological organism to the biomaterial. Since the approach for the deposition of the Ta coatings involves changing the structure and properties of materials at both the micro- and nanoscales, it is advisable to assess changes in the structure and properties at these levels. Such instrumental research methods as atomic force microscopy (AFM) and nanoindentation (NI) allow studying the surface properties of the films of tantalum and its compounds at the nanoscale, as well as to evaluate the nature of their changes (roughness, friction coefficient) in the biological medium and estimate the possibility of using these nanocoatings as biocompatible materials [37,38,39,40].

The aim of the present work was to study the physical, mechanical, and tribological properties of nanostructured tantalum oxynitride coatings on the stainless steel substrates. This complex of characteristics helps to assess the performance characteristics of coatings more accurately than the traditionally used microhardness. Estimation of such a complex is vital for the production of devices for various medical applications.

## 2. Materials and Methods

The coatings of Ta, Ta_2_O_5_, TaN, and TaON were deposited on the polished stainless steel (type is 316 L SS) substrates using reactive direct current planar magnetron sputtering. Deposition process was performed on the experimental set-up (KhNU and NSC KIPT NASU, Kharkov, Ukraine) [41,42]. The physical and chemical processes in plasma were investigated during the reactive magnetron deposition of tantalum oxynitride [43]. Prior to the deposition, substrates were cleaned in an ultrasonic bath, then the ion cleaning was performed (Hall type ion source) in argon atmosphere (pressure was 6.65 × 10^−2^ Pa, ion acceleration voltage—3 kW, ion source current—100 mA) during 5 min. Then, the substrates were placed in a chamber pumped to a residual vacuum of less than 10^−3^ Pa. The ion cleaning was performed (Hall type ion source) in argon atmosphere (pressure was 6.65 × 10^−2^ Pa, ion acceleration voltage—3 kW, ion source current—100 mA) during 5 min. Then the deposition of the coatings of Ta, Ta_2_O_5_, TaN, TaON of about 1 μm thickness was conducted. The tantalum target with a diameter of 170 mm was used. The magnetron discharge power was 4–5 kW. The distance between magnetron and samples was about 30 cm. The feature of this system was the additional oxygen activation by discharge plasma source induction. The oxygen was supplied through a plasma source for activation. The deposition parameters of the coatings are summarized in Table 1. These deposition modes were selected after optimization of the spraying technology carried out in [42,43].

The morphology of the coatings was evaluated by the AFM Dimension FastScan (Bruker, Santa Barbara, CA, USA) in PeakForce QNM (Quantitative NanoMechanics, Bruker, Santa Barbara, CA, USA) regime with CSG10_SS (Micromasch, Tallinn, Estonia) cantilevers. The study of the microstructure and elemental composition of the samples was carried out on the JSM7001F (JEOL, Tokyo, Japan) scanning electron microscope (SEM) equipped with the X-ray energy dispersive microanalysis probe system INCA ENERGY 350 (Oxford Instruments, Abingdon, Oxfordshire, UK) at x10,000 magnification. The operating voltage and probe current were 20 KV and 5 nA, respectively. The working distance was 10 mm. The microstructure was analyzed in the secondary electron mode (SEI mode).

The phase compositions were studied by X-ray phase analysis (XRD) on a DRON-4-07 (LNPO “Burevestnik”, Saint-Petersburg, Russia) unit in copper radiation. To analyze the amorphous and crystal structure formation, coatings were annealed at a temperature of 700 °C for 15 min and one hour in air in a Nabertherm GmbH L5 /13/ B180 furnace.

The method for the investigation of the friction coefficient (CoF) using AFM is based on measuring the twist angle of the silicon cantilever of the probe around its axis under the action of friction between the surface and the tip. It is described in detail in the paper by Chizhik et al. [44]. In the present research, we used NCS 11A silicon cantilevers (Mikromasch, Tallinn, Estonia) with the stiffness 3 N/m to determine the CoF using AFM NT-206 in a “sliding” mode. During the tests on the five different 20 × 4 μm areas and 256 × 50 points, the probe load was 0.005 μN, and the friction speed was 4.9 μm/s. The radius of curvature of the cantilever was increased to 100 nm by scanning at high loads on the silicon surface in order to prevent these changes during research.

**Figure 1:**
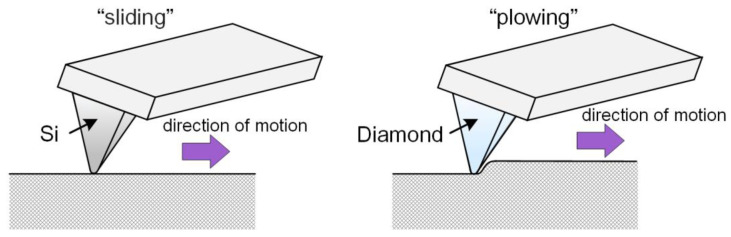
The principle scheme of friction mechanisms: “sliding” and “plowing”.

The CoF in a “plowing” mode and the wear of the coatings were studied using a Dimension FastScan AFM in the Contact Mode using a diamond probe on silicon console of D300 type (SCDprobes, Tallinn, Estonia) with an initial tip radius of curvature of 33 nm The stiffness of cantilevers was 13.84 N·m^−1^. During the tests, the normal load of 1.164 μN (calculated = 0.6 V) per probe was controlled. The process parameters, which were kept constant, were as follows: the scanning area 20 × 4 μm, 100 cycles, 256 × 256 points, friction speed 2.0 μm/s. The movement of the probe on the surface was reciprocating. Thus, the use of silicon and diamond probes with different loads allowed exploring the surface of tantalum coatings by various friction mechanisms (Figure 1). At the “sliding” mode, the action of adhesive forces was influenced on the friction coefficient, and the “plowing” mode characterized the strength properties of the material during friction.

The friction force (*F*) was recorded separately in the forward and in the reverse scanning. In the processing program, the image of the reverse scanning was subtracted from the obtained image of the forward scanning, and thus the average value of the friction force was determined. The mechanical stresses in the contact zone of the AFM probe with the coating surface (contact pressure) were determined using the AMES (Advanced Mechanical Engineering Solutions) contact stress calculator [45,46], setting the values of the radius of curvature of the probe, the elastic modulus of the coatings, and the probe. The value of the specific volumetric wear k_v_ was calculated as the ratio of the volume of worn material (*V*) to the normal load (*L*) and the indenter path length along the sample (*S*) and expressed in m^3^/N∙m:

*k_v_* = *V*/(*L*·*S*) (1)

The volume of wear *V* was estimated by the cross-sectional area and the perimeter of the wear track [47].

The thickness of the coatings was determined via AFM scanning of cross sections of coatings–substrate obtained after cooling samples into the fluid nitrogen during 10 min and fracture of cooled samples.

The microhardness (*H*) and the elastic modulus (*E*) were measured with using Hysitron 750 Ubi nanoindentation device (Bruker, Minneapolis, MN, USA). The radius of curvature of the diamond Berkovich indenter was 150 nm. For each sample, 9 curves were obtained at the load of 2000 μN. The indentation depth (*h*) into the coatings was 50–80 nm. The radius of the tip was calibrated by a set of indentations with increasing load into a surface of standard fused silica sample.

## 3. Results and Discussion

### 3.1. Elemental Analysis of Coatings

**Figure 2:**
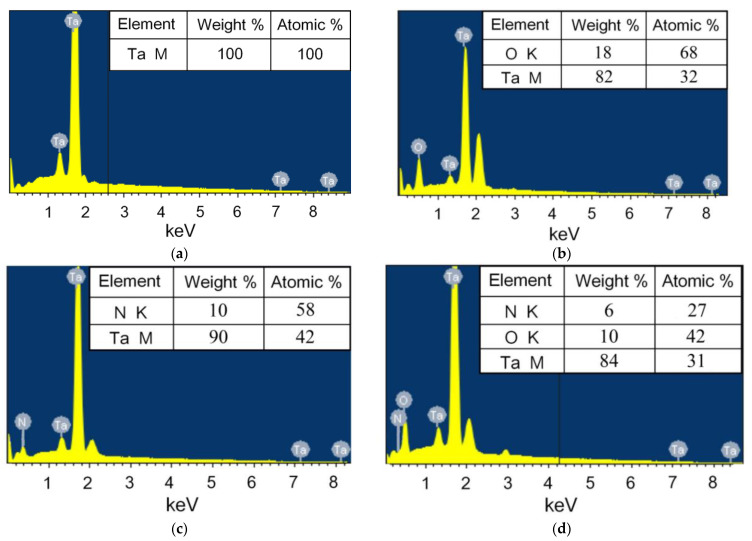
EDX spectra of nanostructured coatings: tantalum (**a**), tantalum oxide (**b**), tantalum nitride (**c**), and tantalum oxynitride (**d**).

**Figure A1:**
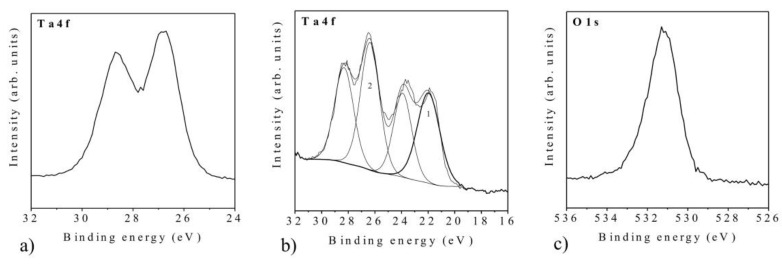
XPS spectra of Ta-based coatings deposited on the steel substrates: Ta^+5^ for Ta_2_O_5_ (**a**), Ta^+5^ and Ta^0^ for Ta/Ta_2_O_5_ (**b**), O1s for Ta_2_O_5_ (**c**) [49].

The EDX spectra (Figure 2) confirm the presence of basic characteristic elements in the films, such as tantalum, oxygen, and nitrogen [48]. The amount of tantalum is close to its atomic content in the compounds Ta_2_O_5_, TaN, and TaON. Except for the main lines of tantalum, the peak at 2.2 KeV corresponds to a secondary line of Ta (Figure 2b–d). Probably, the peak intensity is related to the texture of different Ta-based coatings. Deviations from the stoichiometric composition of coatings can be associated with some method accuracy. Non-uniformity of a scan coating surface and the presence of a small amount of other elements in the spectrum, for example Ar, may affect the error in the results normalizing. Previously carried out research [49] demonstrated the stoichiometric composition of the tested coatings. X-ray photoelectron spectroscopy was carried out using ESCALAB MkII (VG Scientific, East Grinstead, UK) with 1486.6 eV Al Kα radiation. Detailed scans were detected for the C1s, O1s, N1s, and Ta4f regions (Figure A1). An error on the binding energy (BE) values was obtained by standard deviation about 0.2 eV. Data analysis was made with a Shirley-type background subtraction, non-linear least-squares curve fitting with Gaussian-Lorentzian peak shapes. The atomic compositions were calculated using peak areas. The compositional analysis of oxide Ta_2_O_5_, oxynitride TaON, and nitride TaN coatings by X-ray photoelectron spectroscopy was performed. The photoelectron spectra of Ta4f, O1s, and N1s coatings were observed [49]. The spectrum included the photoelectron lines for Ta (4f7/2) and Ta (4f5/2). The Ta^+5^ signals were detected at binding energies 26.8 eV and 28.7 eV. The O1s high-resolution spectra demonstrated the peak at binding energy position *E_b_* = 530.9 eV, associated with Ta-O chemical bond. The N1s peak was detected at binding energy *E_b_* = 396.2 eV, associated with Ta-N chemical bond. This peak is generally considered to be the evidence for replacement of O atoms by N atoms in Ta_2_O_5_ crystal lattices [50]. In addition, a slight N1s peak at binding energy *E_b_* = 398.0 eV was corresponded to Ta-N-O chemical bonds [51]. All binding energies of the high resolution spectra were calibrated with the C1s binding energy of 285.0 eV.

**Figure A2:**
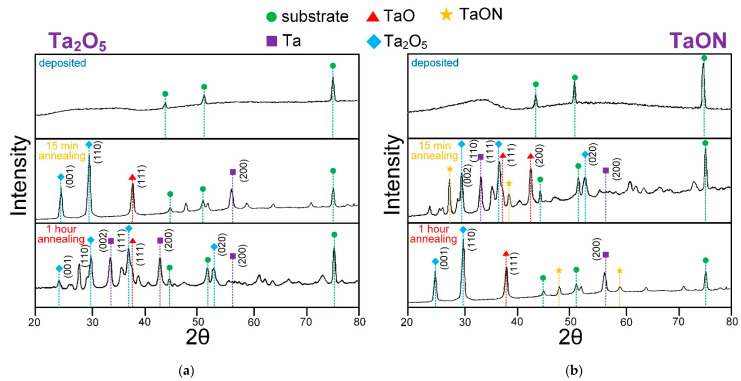
Diffraction X-ray profiles of the Ta_2_O_5_ (**a**) and TaON (**b**) coatings after application and annealing at a temperature of 973 K for 15 min and 1 h.

XRD spectra of as-deposited and annealed Ta_2_O_5_ and TaON coatings were analyzed (Figure A2). According to the XRD data, the amorphous nature of the as deposited Ta_2_O_5_ coatings was confirmed. Changes were detected for the Ta_2_0_5_ coatings after the treatment at 700 °C for 15 min, as confirmed by the XRD pattern peaks which became sharper and more intense (Figure A2). The increase in the crystallinity of the coatings as a function of thermal treatment temperature was detected. After 15 min annealing at a temperature of 700 °C, the peaks typical for the formation of the crystal structure of Ta_2_O_5_ (001), (110), (111), as well as the peaks typical for Ta (200), were clearly identified. Further heating for 1 h led to an increase in the intensity of main peaks and the appearance of a new one (020) in the angular range of 24–72 degrees (2θ). In the case of TaON, characteristic peaks of oxynitride at angles of 27°, 33°, 36°, 38°, as well as spectra associated with the formation of TaON structure at angles in the range of 61–63 degrees (2θ) were detected after 15 min of annealing. In addition, some characteristic peaks of the nitride structure (110), (111), (220) were revealed. Subsequent annealing for an hour was resulted in the further formation of the oxynitride structure and increase in the characteristic peaks at the angles of 23°, 37°, 47°, and 67°.

### 3.2. The Thickness of the Coatings and Fracture Microstructure

**Figure 3:**
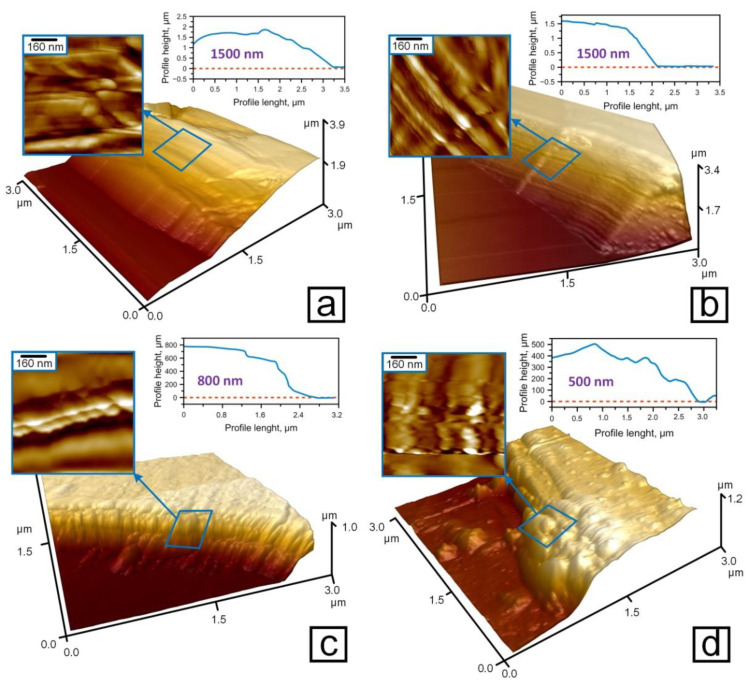
AFM-images of nanostructured coatings fractures: tantalum oxide (**a**), tantalum oxynitride (**b**), tantalum nitride (**c**), and tantalum (**d**).

**Figure A3:**
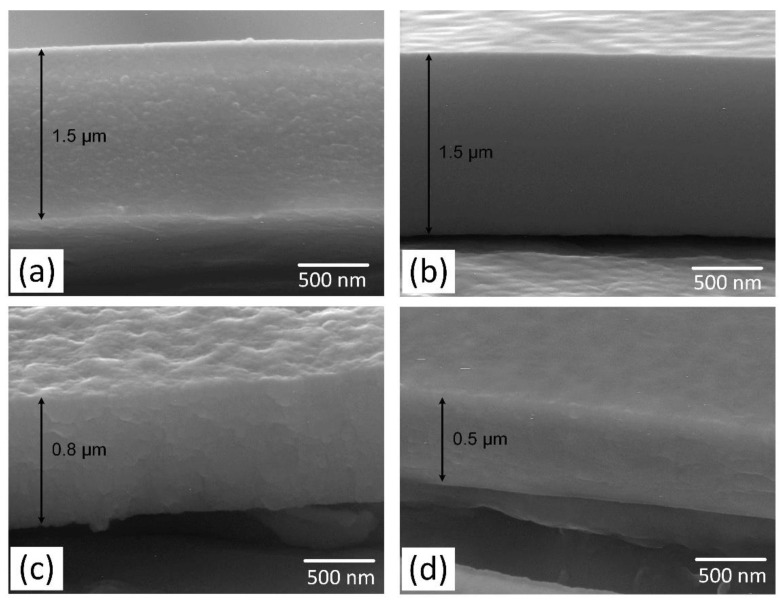
SEM-images of nanostructured coatings cross-sections: tantalum oxide (**a**), tantalum oxynitride (**b**), tantalum nitride (**c**), tantalum (**d**).

AFM images of fractures of the investigated coatings of tantalum compounds on steel and their surface profiles are demonstrated on the Figure 3. According to these profiles, the values of the thickness for the coatings were as follows: Ta_2_O_5_ and TaON—1500 nm, TaN—800 nm, and Ta—500 nm, which is confirmed by the SEM data (Figure A3). In addition, the fracture sites of coatings allow a qualitative assessment of the brittleness of coatings. Thus, the fracture sites of Ta_2_O_5_ and TaN coatings have in their microstructure significant fragments of a “columnar” texture (arrows in Figure 3a,c), which are typical for more brittle fracture. The microstructure of TaON coatings shows strips with a thickness of 20–40 nm (arrows in Figure 3b). The layered Ta and fracture sites coatings consist of nanosized grains of 40 nm in diameter (arrows in Figure 3d). In the case of fracture, the grains are arranged in rows under the action of deformation. These structures are typical for the more plastic materials, according to the plasticity values determined by NI: the highest value for Ta (59.2%), the high for TaON (52.3%), the middle for TaN (42.3%), and Ta_2_O_5_ has the lowest *η* (27.2%).

### 3.3. The Surface Microstructure of Coatings

**Figure 4:**
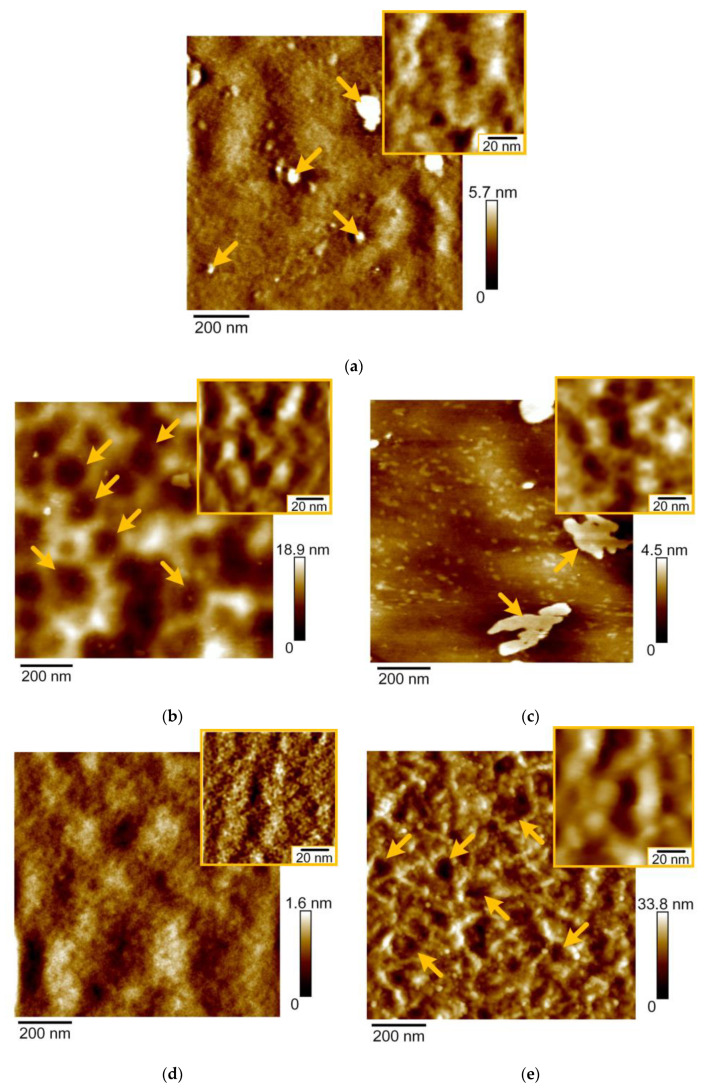
AFM-images of stainless steel (**a**) and the coatings: tantalum (**b**), tantalum oxide (**c**), tantalum oxynitride (**d**), and tantalum nitride (**e**).

In the process of magnetron sputtering, the mechanism of growth is determined by the balance of the energy of the substrate surface, the deposited material, the energy of the material-substrate interface, and the energy of elastic stresses in the growing film. High-resolution AFM is needed to reveal the surface morphology and roughness of smooth amorphous coatings on polished substrates. According to AFM-images, the polished surface of the stainless steel has a microstructure with irregularities and protruding particles of alloying phases of 20–200 nm in diameter (arrows Figure 4a). The arithmetic mean roughness (*R_a_*) for the steel surface on the area of 4 × 4 µm was 3.8 nm.

Surface uniformity was increased after tantalum was deposited on the steel surface. On the area of 1 × 1 µm, Ta coatings have the cellular surface with ribbings that are limitative for the cells (arrows Figure 4b). The ribbings have granular microstructure with the diameter of grains of 20 nm that are shown on the area of 60 × 60 nm scanning field (inset Figure 4b). *R_a_* of Ta coatings on the area 4 × 4 µm was 4.8 nm.

Flat islets with the height of 4–6 nm and the size of 100–500 nm appeared on the Ta_2_O_5_ coatings in the process of the sputtering (arrows Figure 4c). These flat islets indicate that a growth mechanism of tantalum oxide is different compared to the one of tantalum films. Ta coatings are characterized by polycrystalline growth, while tantalum oxide is characterized by layer-by-layer growth. On the area of 60 × 60 nm (inset Figure 4c), Ta_2_O_5_ coatings have granular microstructure with the diameter of grains of 5–20 nm. *R_a_* of Ta_2_O_5_ coatings on the area 4 × 4 µm was 4.2 nm.

The surface of TaON coatings is characterized by a granular structure with the small crystallites of 5 nm in diameter (inset Figure 4d). The surface of TaN coatings consists of grains of 20 nm in diameter (inset Figure 4e). These grains assembled into chains, which formed the edges of cells 200 nm in size (arrows Figure 4e). *R_a_* values of TaN and TaON coatings on the area 4 × 4 µm were 9.5 and 5.6 nm, respectively.

The obtained morphology of the coatings of tantalum and its compounds makes it possible to explain the numerical values of the roughness.

The obtained results on AFM microstructure of the coatings of tantalum compounds are consistent with the previously published studies of Ta [52] and TaN [53] coatings which had a granular structure. The grain size is significantly smaller and is close to calculations in the research of Alishahi et al. [54].

### 3.4. The Mechanical Properties of Coatings

**Figure 5:**
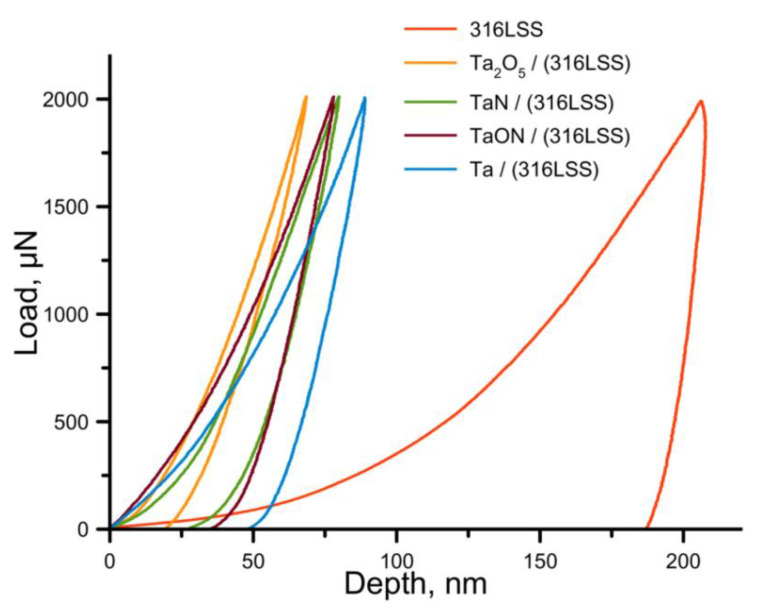
The dependence curves of load on the indentation depth *h*.

The diagrams, showing dependence of the indentation depth on indentation load for the coatings and the steel are demonstrated in the Figure 5. The shape of the curves and their position according to the Y axis show the distribution of coatings by *H*: the closer the curve is to the Y axis, the harder the material is. The area bounded by the curves of the approach–retraction characterizes the plastic deformation. According to the curves the significant difference in mechanical properties of coatings and substrate is visible.

It was shown that the values of *H* and *E* of stainless steel 316 L SS were determined as 1.7 ± 0.2 and 150.0 ± 10.0 GPa, respectively. Plasticity according to the indentation curve area was the heighest—91.4% and *H*/*E*—the lowest—was 0.01. Metallic Ta coatings are characterized by the lowest *H* of 8.3 ± 0.2 GPa. Nonmetallic addition of O and N increased the value of *H* to 10.0 ± 0.3 GPa for TaN, 13.3 ± 0.6 GPa for TaON, and 16.0 ± 3.5 GPa for Ta_2_O_5_. The values of the elastic moduli of four studied coatings on the stainless steel substrates are about 158.0 GPa. These values are close to *E* of 156 GPa and *H* of 10 GPa for bulk pure Ta, obtained and described by Kommel et al. [55]. After the coatings’ deposition, the strength of the steel substrate increased and *H*/*E* allows to estimate by how much: 0.05 for Ta, 0.06 for TaN, 0.08 for TaON, and 0.10 for Ta_2_O_5_. The coatings on stainless steel substrates (304 SS), formed via reactive magnetron sputtering by the authors of [56], showed lower mechanical properties for coatings of Ta compounds: for Ta_2_O_5_—*H* of 5.8 GPa, *E* of 135 GPa, and *H*/*E* of 0.049 and for TaON—*H* of 7.5 GPa, *E* of 119 GPa, and *H*/*E* of 0.056. The reason of the underestimated values of *H* and *E* in [56] may be the lower content of Ta in the coatings in comparison to those studied in this work. In [57], for coatings of 700–100 nm thickness, the values of *E* were 127 GPa for Ta and 108 GPa for Ta_2_O_5_, and the values of *H*—6.8 GPa for Ta and 8.4 GPa for Ta_2_O_5_. The somewhat underestimated values of the characteristics can be explained by the excessive indentation depth and the influence of the substrate.

**Figure 6:**
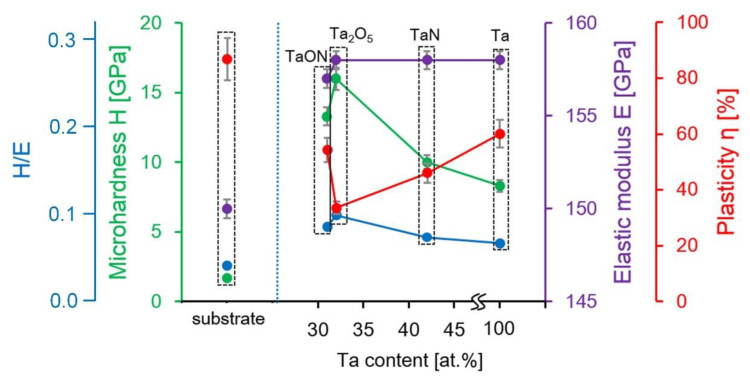
The dependences of *H*/*E*, *H*, *E* and *η* of Ta based coatings on the Ta atomic content.

The dependences of the mechanical properties of investigated coatings on the atomic content of Ta are shown in Figure 6. The higher oxygen and nitrogen content in the coatings based on Ta, the greater the microhardness of the surface. This tendency is associated with the introduction of oxygen and nitrogen atoms into the tantalum crystal structure, what leads to compressive residual stress in the coatings [58]. In addition, the authors of work [59] found that the higher the oxygen content, the higher the average hardness.

On the base of the dependence of *E* and *H* on the atomic content of Ta coatings on the steel (Figure 6), the Ta content in the coatings does not affect the elastic modulus but affect the hardness. Such effect was described in [55] where two existing polytypes of tantalum α-Ta and β-Ta showed the same *E* of 188 GPa and the different *H*: 10 GPa for α-Ta and 18 GPa for β-Ta. The discrepancy in the values of *E* and *H* can be explained by the film thickness (of 100 and 300 nm), which is usually higher for thin films.

Taking into account the whole set of physical and mechanical characteristics of Ta coatings and its compounds, TaON is the optimal coating, which simultaneously has sufficient hardness and high plasticity.

### 3.5. The Tribological Characteristics

**Figure 7:**
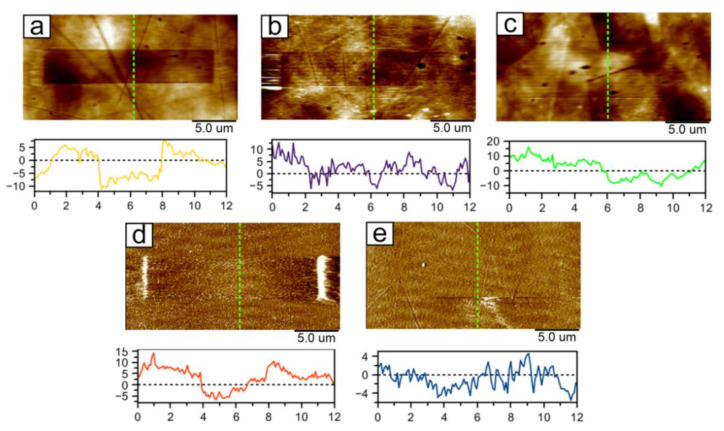
AFM-images of wear test results at loading of 1.164 μN, speed of 2 μm/s, and 100 cycles on the steel substrate and the tantalum coatings: steel (**a**), Ta_2_O_5_ (**b**), TaON (**c**), TaN (**d**), Ta (**e**).

The results of friction and wear tests in the “plowing” regime are shown in Figure 7. Wear tracks on TaON and Ta coatings are visible only in the Friction regime. These tracks show the efficacy of Ta based coatings of wear protection of steel. Wear tracks on TaN (Figure 7d) and Ta (Figure 7e) coatings are visible only in the PeakForce Error data type. Since PeakForce QNM uses Peak Force as the feedback signal, the PeakForce Error data type is essentially the Peak Force Setpoint with the error. It is recorded simultaneously with the topography. In this mode, the boundaries of the wear mark are better visible. We had to use the error signal as the wear on these materials was very low. The topography mode did not allow it to be visualized against the background of scratches and protruding microparticles. Moreover, the error signal showed the contours of the worn material at the boundaries of the wear marks located across the scanning direction due to a significant change in the Peak Force Setpoint in this scanning area.

**Figure 8:**
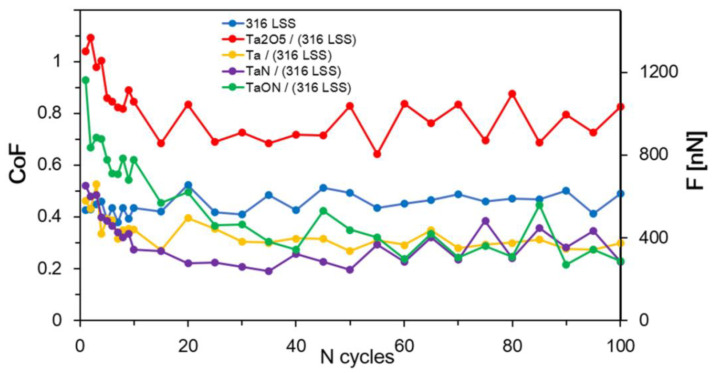
Dependence of the obtained friction coefficients on the number of friction cycles of the studied coatings on the steel substrates.

The dependences of the CoF and *F* of the coatings during the wear in the “plowing” mode to the number of friction cycles are presented in Figure 8. Each point in the obtained diagrams is averaged over 50 scanning lines. “Teeth” in values of CoF are explained by position of the probe in each cycle with respect to the surface and AFM- photodetector: «upper–down» or «down–upper». The higher the value of CoF, the larger the “part” of probe twisting per when changing the position of the probe at reciprocating motion. CoF values were decreased after 15 cycles; it is explained by «breaking-in» to expressed in the change in the subnanometer layer of the material under the tribological load from the start of scanning and its uniform distribution over the surface. CoF in steady-state for steel was 0.448. The coatings TaON, Ta, and TaN allow decreasing CoF of the surface down to 0.444, 0.336, and 0.308, respectively (Figure 8). The deposition of Ta_2_O_5_ coatings on the steel substrate increases CoF to 0.780.

**Figure 9:**
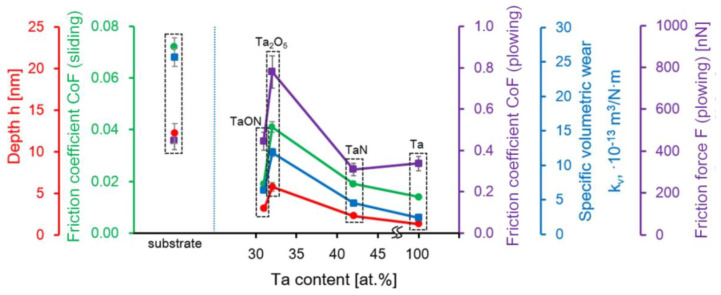
The dependences of *h*, CoF (sliding), CoF (plowing), *k_v_*, and *F* of Ta based coatings on the Ta atomic content.

Specific volumetric wear allows comparing coatings of tantalum and its compounds with other materials quantitatively. All the tantalum coatings under research are capable of reducing the wear of steel (25.4 × 10^−13^ m^3^/N∙m) more than twice (Figure 9). The minimum value of specific volumetric wear were recorded for the Ta coatings—2.1 × 10^−13^ m^3^/N∙m. TaN and TaON showed the middle values—4.2 × 10^−13^ and 6.1 × 10^−13^ m^3^/N∙m. The minimum wear were determined for Ta_2_O_5_ coatings—11.6 × 10^−13^ m^3^/N∙m. The dependences of specific volumetric wear on Ta atomic content in coatings are in good agreement with their CoF (Figure 9). The “plowing” mode allows assessing the real strength properties of the material during the friction.

The influence of the adhesive forces is better characterized by the “sliding” mode [60,61]. The determined CoF for stainless steel in the “sliding” mode is 0.072 (Figure 9). After the deposition of nanostructured tantalum coatings, the CoF decreases to 0.014 (Ta) and 0.019 (TaN and TaON). CoF of Ta_2_O_5_ coatings in the “sliding” mode is 0.041. The dependences of CoF in the “sliding” and “plowing” modes on Ta atomic content in coatings have a similar behavior (Figure 9).

The better tribological properties of Ta coatings can be explained by its microstructure and plasticity of the coating. The low CoF values of the oxynitride film can be explained by the high roughness, and the high values of tantalum oxide, by the low roughness. Microhardness is often considered as the main characteristic to predict the wear. The Ta_2_O_5_ coatings with the highest *H* of 18 GPa and *R_a_* of 4.2 nm showed the weakest nanotribological properties. This result can be explained by the significantly different mechanism of wear in the environment of nanofriction contact from usual classical mechanism of macrocontact.

The authors of [35] obtained TaNx coatings by the high-frequency magnetron sputtering method. Tribological researches were carried out by the nanoindentation method using a Berkovich diamond tip at a load of 5 μN. The average friction coefficient was ~0.18. The significant difference between CoF in [35] and those obtained by us for TaN (0.30) is explained by the low applied load during tribological tests of AFM. In [36], tantalum nitride films were synthesized on silicon using magnetron sputtering. A pin-on-disk (alumina ball) tribometer at 1 N load was used to obtain the coefficient of friction and the wear rate of these films. It was found that 13% N_2_ films have the highest hardness, a low coefficient of friction (~0.6), and a reduced wear rate (7.09 × 10^−15^ m^3^/N·m). The discrepancy in the values of CoF is associated with an increased load and a significantly larger contact area in [35]. Wear during AFM tests is overestimated due to high mechanical stresses in the contact associated with the small radius of the tip, wherein AFM-wear makes it possible to accurately determine the difference between all compared coatings.

The used normal forces about 1 µN and a nanoscale radius of the probe (8–76 nm) lead to the creation on the surface of coatings of significant contour pressure (or mechanical stress in contact, contact pressure) of 15–65 GPa. This value is many times higher than the level of contact stresses at macro-tribotests of about 1.3–1.6 GPa. The localization of maximal mechanical stresses at nanometers depth and displacement of the material from the surface by atomic layers gives a different mechanism of the friction process under conditions of microcontact. The high *η* of Ta coatings allow light moving of atomic layer of materials under the probe acting.

The TaON coatings were chosen according to the strength properties because of the rather high values of *H* and simultaneously high *η* and *H*/*E* value of 0.08, then according to tribological characteristics—Ta, TaN, and TaON.

## 4. Conclusions

Nanostructured films of tantalum compounds were deposited on the stainless steel substrates by magnetron sputtering. It was determined that the microstructure of coatings depends on the elemental composition. All tantalum based coatings are characterized by a granular structure. Depending on the composition of the coatings, the grains vary in size from 5 to 20 nm. In some cases (Ta and TaN), the grains associate into cells. All obtained coatings have low roughness values.

The best combination of properties among the studied coatings have TaN (*H* of 10.0 GPa, *E* of 158.0 GPa and *H*/*E* = 0.06) and TaON (*H* of 13.3 GPa, *E* of 157.0 GPa and *H*/*E* = 0.08).

The tribological characteristics of obtained coatings were: TaN—CoF of 0.019 in the ”sliding“ mode and 0.308 in the ”plowing” mode, specific volumetric wear of 4.2 × 10^−13^ m^3^/N∙m, TaON—CoF of 0.019 in the ”sliding” mode and 0.444 in the ”plowing” mode, and specific volumetric wear of 6.1 × 10^−13^ m^3^/N∙m. Thus, deposition of TaN decreases specific volumetric wear of steel by more than 6 times, TaON—by more than 4 times. These tribological characteristics allow reducing of platelets on the surface of stents, and thereby, preventing the formation of a blood clot.

Thus, TaN and TaON coatings, which have a special complex of mechanical and tribological properties, can be used as upper layer for stainless steel stents.

## Figures and Tables

**Table 1:** The parameters of the magnetron sputtering for the coatings of tantalum and its compounds.

Type of Coating	Gas Pressure P [Pa]	Magnetron Voltage U_m_ [V]	Magnetron Current I_m_ [A]	Time of Deposition [min]	Gas Mass Flow Rate Q [cm^3^/min]
Ta	1 × 10^−1^ (Ar)	495	6.6	30	-
Ta_2_O_5_	1.3 × 10^−1^ (general)	500	6.4	20	25 (O_2_)
TaN	1.2 × 10^−^^1^ (N_2_)	800	3.4	60	95 (N_2_)
TaON	1.5 × 10^−1^ (general)	620	4.0	30	10 (O_2_) 45 (N_2_)
